# Ultra-high gradient performance 3-Tesla MRI for super-fast and high-quality prostate imaging: initial experience

**DOI:** 10.1186/s13244-024-01862-x

**Published:** 2024-11-29

**Authors:** Leon M. Bischoff, Christoph Endler, Philipp Krausewitz, Joerg Ellinger, Niklas Klümper, Alexander Isaak, Narine Mesropyan, Dmitrij Kravchenko, Sebastian Nowak, Daniel Kuetting, Alois M. Sprinkart, Petra Mürtz, Claus C. Pieper, Ulrike Attenberger, Julian A. Luetkens

**Affiliations:** 1 Department of Diagnostic and Interventional Radiology, University Hospital Bonn, Bonn, Germany; 2 Quantitative Imaging Lab Bonn (QILaB), University Hospital Bonn, Bonn, Germany; 3 Department of Urology, University Hospital Bonn, Bonn, Germany

**Keywords:** Multiparametric MRI, Biparametric MRI, Prostate cancer, PI-RADS, Ultra-high gradient strength

## Abstract

**Objectives:**

To implement and evaluate a super-fast and high-quality biparametric MRI (bpMRI) protocol for prostate imaging acquired at a new ultra-high gradient 3.0-T MRI system.

**Methods:**

Participants with clinically suspected prostate cancer prospectively underwent a multiparametric MRI (mpMRI) on a new 3.0-T MRI scanner (maximum gradient strength: 200 mT/m, maximum slew rate: 200 T/m/s). The bpMRI protocol was extracted from the full mpMRI protocol, including axial T2-weighted and diffusion-weighted (DWI) sequences (b0/800, b1500). Overall image quality was rated by two readers on a five-point Likert scale from (1) non-diagnostic to (5) excellent. PI-RADS 2.1 scores were assessed by three readers separately for the bpMRI and mpMRI protocols. Cohen’s and Fleiss’ κ were calculated for PI-RADS agreement between protocols and interrater reliability between readers, respectively.

**Results:**

Seventy-seven male participants (mean age, 66 ± 8 years) were included. Acquisition time of the bpMRI protocol was reduced by 62% (bpMRI: 5 min, 33 ± 21 s; mpMRI: 14 min, 50 ± 42 s). The bpMRI protocol showed excellent overall image quality for both the T2-weighted (median score both readers: 5 [IQR: 4–5]) and DWI (b1500) sequence (median score reader 1: 4 [IQR: 4–5]; reader 2: 4 [IQR: 4–4]). PI-RADS score agreement between protocols was excellent (Cohen’s κ range: 0.91–0.95 [95% CI: 0.89, 0.99]) with an overall good interrater reliability (Fleiss’ κ, 0.86 [95% CI: 0.80, 0.92]).

**Conclusion:**

Ultra-high gradient MRI allows the establishment of a high-quality and rapidly acquired bpMRI with high PI-RADS agreement to a full mpMRI protocol.

**Trials registration:**

Clinicaltrials.gov, NCT06244680, Registered 06 February 2024, retrospectively registered, https://classic.clinicaltrials.gov/ct2/show/NCT06244680.

**Critical relevance statement:**

A novel 3.0-Tesla MRI system with an ultra-high gradient performance enabled high-quality biparametric prostate MRI in 5.5 min while achieving excellent PI-RADS agreement with a standard multiparametric protocol.

**Key Points:**

- Multi- and biparametric prostate MRIs were prospectively acquired utilizing a maximum gradient of 200 mT/m.
- Super-fast biparametric MRIs showed excellent image quality and had high PI-RADS agreement with multiparametric MRIs.
- Implementation of high gradient MRI in clinical routine allows accelerated and high-quality biparametric prostate examinations.

**Graphical Abstract:**

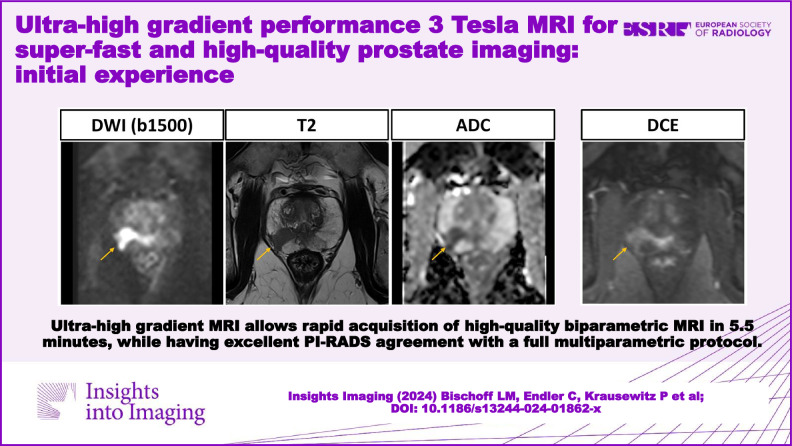

## Introduction

Multiparametric MRI (mpMRI) of the prostate has become an important non-invasive diagnostic tool for the assessment of suspected prostate cancer and is considered the baseline for MRI-targeted biopsy [1, 2]. As the incidence of prostate cancer is high, with an estimated 290,000 new cases in 2023 in the United States alone [3], the need for widespread and high-quality provision of prostate MRI will continue to rise. However, current clinical MRI protocols are long, with acquisition times of > 30 min, potentially limiting the absolute number of MRI scans. According to the guidelines of the Prostate Imaging Reporting and Data System (PI-RADS), a sufficient mpMRI protocol must include diffusion-weighted imaging (DWI), T2-weighted (T2w) imaging, dynamic contrast-enhanced imaging, and T1-weighted pre- and post-contrast images [4, 5]. Different approaches have been developed to shorten the protocol itself or to accelerate acquisition times. For instance, a significant reduction of T2w-sequence acquisition times was achieved by employing deep learning reconstruction methods [6, 7] or the utilization of advanced compressed sensing [8]. Different studies showed equal performance of biparametric MRI (bpMRI) protocols compared to the standard multiparametric protocols, effectively reducing acquisition times [9–11]. This was done by focusing only on DWI and T2w imaging while omitting the dynamic contrast-enhanced sequence and T1-weighted sequences, as the additional diagnostic value of these is controversial [12].

Recent developments in MRI scanner hardware have allowed for ultra-high diffusion gradient strengths (up to 500 mT/m), thus reducing echo times and acquisition times by establishing faster diffusion gradients [13, 14]. Furthermore, these gradients can image at smaller scales with a higher signal-to-noise ratio, consecutively enhancing sensitivity for the detection of tissue microstructures by a higher spatial resolution [15, 16]. Due to the experimental nature of these gradients, this technique has only been investigated in research settings in healthy volunteers [17]. However, recently introduced high gradient 3.0-T MRI technology with clinical FDA clearance gives the opportunity to evaluate high gradient imaging in whole-body MRI, including prostate imaging.

Therefore, our study aimed to implement a super-fast and high-quality abbreviated bpMRI protocol for patients with suspicion of prostate cancer using ultra-high gradient DWI. Aside from assessing acquisition times and image quality, the main objective of this study was to compare PI-RADS scores in the bpMRI protocol with the full mpMRI protocol.

## Materials and methods

### Study population

This prospective study was approved by the institutional review committee of the University Hospital Bonn (Clinicaltrials.gov: NCT06244680) and followed the principles of the Declaration of Helsinki from 2013. After obtaining written informed consent, male participants with suspicion of prostate cancer, defined by elevated levels of prostate-specific antigen (> 4 ng/mL), suspicious digital rectal and/or transrectal ultrasound exams, were continuously and consecutively enrolled in this study between September and November 2023. Participants with general contraindications for MRI at 3.0 T (e.g., total hip replacements of both sides) or contraindication for administration of gadolinium-based contrast media, as well as severe claustrophobia, were excluded from the study.

### Image acquisition

All examinations were performed on a 3.0-T MRI scanner (Siemens MAGNETOM Cima.X, Siemens Healthineers) using an additional anterior phased array coil with 18 channels for signal reception. The whole-body MRI system employs a maximum gradient strength of 200 mT/m and a maximum slew rate of 200 T/m/s, whereby the maximum gradient amplitude might be limited due to adverse effects on the heart, e.g., cardiac stimulation. Participants with no contraindications received 1 mL hyoscine butylbromide (Butylscopolamin 20 mg/mL, Panpharma) once prior to MRI to reduce bowel peristalsis. The contrast agent gadoteric acid (Clariscan, GE Healthcare) was used for post-contrast T1-weighted sequences. A full mpMRI protocol was acquired as the reference standard, consisting of an axial, sagittal and coronal T2w turbo spin-echo sequence, an axial DWI sequence with b-values of 0 and 800 s/mm^2^ (the apparent diffusion coefficient map was calculated from these b-values), an axial DWI sequence with a b-value of 1500 s/mm^2^, a T1-weighted turbo spin-echo Dixon sequence pre and post-contrast media administration, and a dynamic contrast enhanced sequence (Time-resolved angiography With Stochastic Trajectories, TWIST). DWI sequences were acquired using echo-planar imaging (EPI) with zonally-magnified oblique multislice acquisition (ZOOM) and employed a restricted maximal gradient strength of 111 mT/m. Vendor-specific, commercially available deep learning techniques for denoising were utilized for reconstructions of T2w and DWI sequences (Deep Resolve Boost, Siemens Healthineers). Additionally, resolution upscaling was used for T2w sequences (Deep Resolve Sharp, Siemens Healthineers). Only the axial T2w sequence and DWI sequences, which were acquired during the mpMRI, were used for the interpretation of the biparametric protocol. Acquisition times per sequence were added to calculate the overall acquisition times of the bpMRI and mpMRI protocols. Detailed sequence parameters are specified in Table 1.

**Table 1:** Sequence parameters of the bi- and multiparametric prostate protocol

	T2w TSE axial*	DWI axial (b0,b800)*	DWI axial (b1500)*	T2w TSE sagittal	T2w TSE coronal	T1w TSE axial	3D T1w DCE
Image matrix	384 × 384	46 × 90	46 × 90	384 × 384	384 × 384	280 × 352	160 × 160
Field of view (mm^2^)	200 × 200	102 × 200	102 × 200	200 × 200	200 × 200	255 × 320	200 × 200
Spatial resolution (mm^3^)	0.5 × 0.5 × 3	2.2 × 2.2 × 3	2.2 × 2.2 × 3	0.5 × 0.5 × 3	0.5 × 0.5 × 3	0.9 × 0.9 × 2.5	1.3 × 1.3 × 3
Slice thickness (mm)	3	3	3	3	3	2.5	3
Standard slice number (*n*)	26	26	26	24	25	88	24
Echo time (ms)	96	54	57	107	107	2.46	1.5
Repetition time (ms)	8560	3200	3200	6990	7130	5.51	4.41
Flip angle (degree)	90	90	90	90	90	10	15
Averages	1	2 (b0), 8 (b800)	7	1	1	1	1
Diffusion gradients	-	4 directions (4-Scan Trace)	4 directions (4-Scan Trace)	-	-	-	-
Bandwidth (Hz/pixel)	200	1684	1684	200	200	592	679
Acquisition time (s)	105	117	104	99	101	70	203
Temporal resolution (s)	-	-	-	-	-	-	4.22
Fat suppression	-	Fat-Sat	Fat-Sat	-	-	Dixon	-
DL denoising	+	+	+	+	+	-	-
DL resolution upscaling	+	-	-	+	+	-	-

*DCE* dynamic contrast enhanced, *DL* deep learning, *DWI* diffusion-weighted imaging, *SPIR* spectral presaturation with inversion recovery, *TSE* turbo spin-echo, *T1w* T1-weighted, *T2w* T2-weighted

* Included in the biparametric protocol

### Qualitative image analysis

Qualitative image rating of the bpMRI protocol for the T2w sequence and both DWI sequences was performed by two radiologists with 3 (L.M.B.) and 12 (J.A.L.) years of experience in prostate MRI on a five-point Likert scale for six different qualitative categories (artifacts, image sharpness, lesion conspicuity, capsule delineation, overall image sharpness, and diagnostic confidence). Grading was defined as follows: 1, non-diagnostic due to extensive artifacts, strongly impaired conspicuity of anatomical structures, and no diagnostic confidence; 2, several artifacts, difficult conspicuity of anatomical structures, and low diagnostic confidence; 3, moderate artifacts, fair conspicuity of anatomical structures, and moderate diagnostic confidence; 4, little artifacts, good conspicuity of anatomical structures, and good diagnostic confidence; 5, no artifacts, excellent conspicuity of anatomical structures, and high diagnostic confidence. The artifacts category included movement artifacts, metal artifacts, adjacent air artifacts of the rectum, and Gibbs Ringing artifacts. The results of both raters were averaged.

### Quantitative image analysis

An equal-sized region of interest (30 mm^2^) was drawn in the healthy peripheral zone of the prostate and in the internal obturator muscle on both the axial T2w sequence and on the DWI (b1500) sequence. The apparent signal-to-noise ratio (signal intensity in the peripheral zone divided by standard deviation of the signal intensity in the muscle) and the apparent contrast-to-noise ratio ((signal intensity of the peripheral zone minus signal intensity of muscle) divided by standard deviation of the signal intensity in the muscle) were calculated as previously described [6].

### PI-RADS assessment and agreement

Three radiologists with 3 (L.M.B.), 12 (J.A.L.), and 8 (C.E.) years of experience in prostate MRI separately read the bpMRI protocol and graded the lesions according to the PI-RADS classification. Only the highest-graded lesion and its respective prostate zone were noted. If there were two distinct lesions with the same high PI-RADS score in both the peripheral and transition zone, both were noted. Participants were presented in a random order. All readers were blinded to any personal and clinical parameters (e.g., name, age, patient history, value of the prostate-specific antigen, clinical examination, and transrectal ultrasound). After a washout period of one month, all readers repeated the same assessment for the mpMRI protocol.

### Statistical analysis

Statistical analysis was conducted by L.M.B. using SPSS (Version 27, IBM Corp.). The sample size of this study was chosen equivalent to previous biparametric prostate MRI studies (18–20). Continuous variables are given as mean ± standard deviation, whereas discrete variables are given as median and interquartile range (IQR) and binary variables as absolute percentages. Both, the agreement of bpMRI and mpMRI PI-RADS scores for the whole prostate, and for the specific zonal distribution (peripheral and transition zone) were assessed by calculation of Cohen’s κ, interpreted as follows: < 0.5 = poor; 0.5–0.75 = moderate; 0.75–0.9 = good; > 0.9 = excellent. Interrater reliability between readers for PI-RADS scores was assessed equivalently with Fleiss’ κ, while interrater reliability between raters for qualitative analysis was assessed with Cohen’s κ with equal interpretation as above. A *p*-value of < 0.05 was considered statistically significant in all cases.

## Results

### Clinical characteristics of participants

**Fig. 1:**
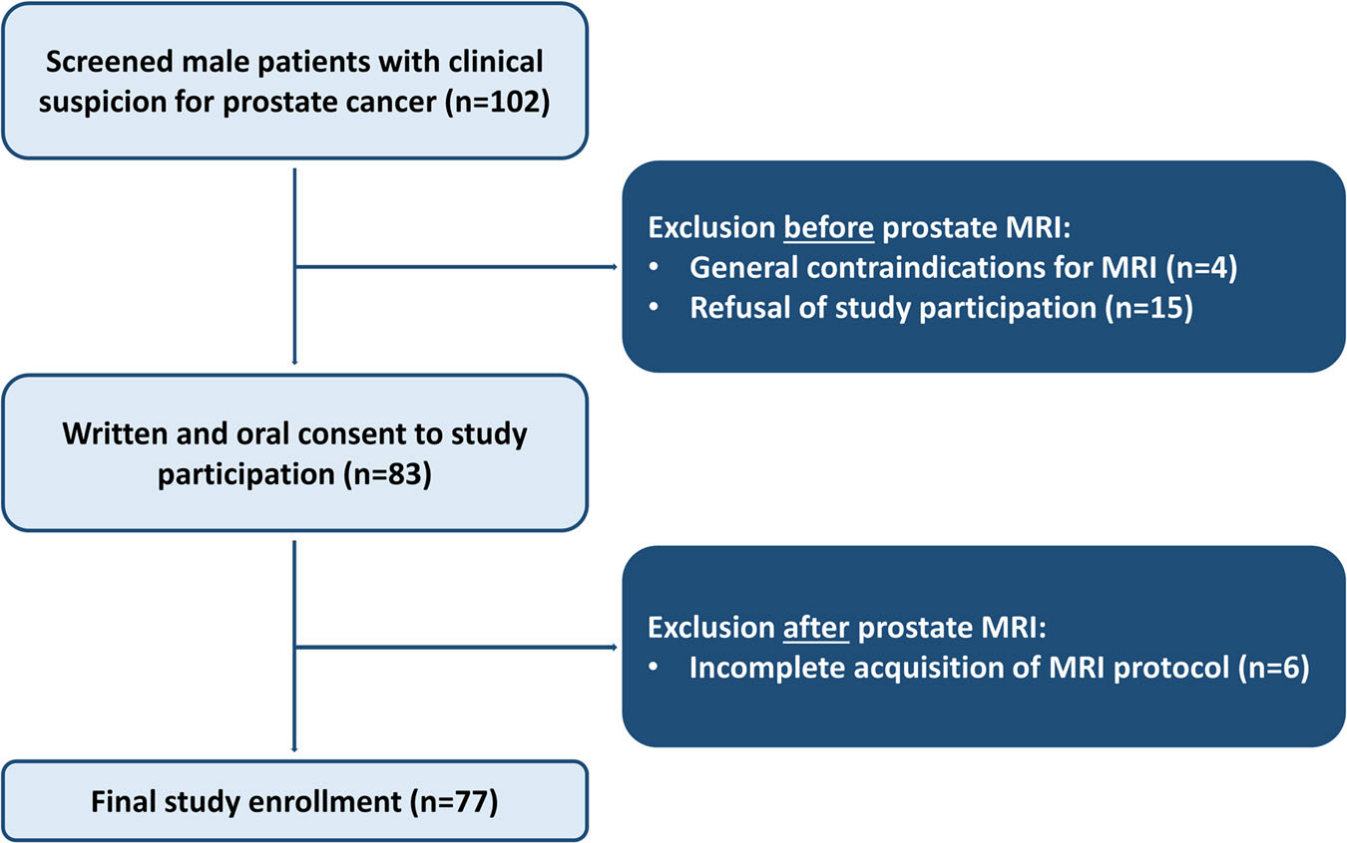
Flowchart of enrolled participants in the study

A total of 77 male participants with a mean age of 66 ± 7 years (range: 53 to 84 years) were included in the study after exclusion of four participants due to general MRI contraindications, fifteen participants due to refusal of study participation and six participants due to incomplete protocol acquisition (Fig. 1). 90% (69/77) of participants had an elevated prostate-specific antigen of > 4 ng/mL, whereas 16% (12/77) had a suspicious digital rectal exam, and 10% (8/77) had a suspicious transrectal ultrasound. 53% (41/77) of participants had an initial PI-RADS score of ≥ 3 and 38% (29/77) underwent subsequent biopsy with confirmed malignancy in 22% (17/77). Median International Society of Uropathology grade was 2 (IQR, 1–2). Detailed clinical characteristics of enrolled participants are given in Table 2. For detailed biopsy results, see Table S1.

**Table 2:** Clinical characteristics of enrolled participants

Variable	Value
No. of participants	77
Age (years)	66 ± 8
Prostate-specific antigen (ng/mL)	8.7 ± 7.2
Suspicious digital rectal exam	12 (16)
Suspicious transrectal ultrasound	8 (10)
Prior biopsy	17 (22)
PI-RADS score	
1	0 (0)
2	36 (47)
3	10 (13)
4	19 (25)
5	12 (16)

Continuous data is reported as mean ± standard deviation and dichotomous data as number of participants with percentages in parentheses

*PI-RADS* Prostate Imaging Reporting and Data System

### Evaluation of image quality

**Fig. 2:**
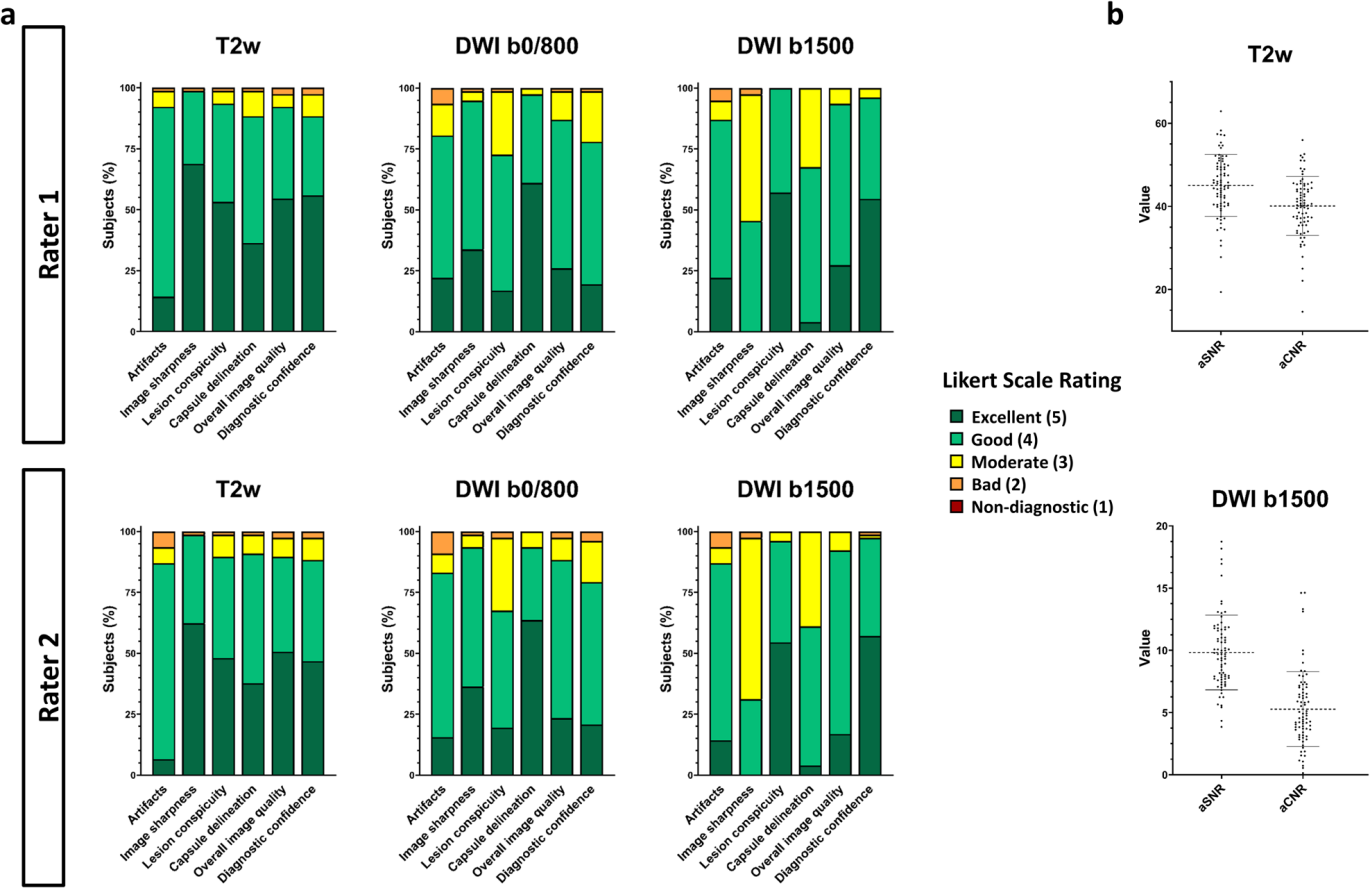
Stacked bar charts show the qualitative Likert ratings in the six categories artifacts, image sharpness, lesion conspicuity, capsule delineation, overall image quality and diagnostic confidence for both raters and each sequence of the biparametric protocol, including a T2-weighted (T2w) sequence and two diffusion-weighted imaging (DWI) sequences with b = 0 and 800 s/mm^2^, and b = 1500 s/mm^2^ (**a**). Scatter plot diagrams show the values of the apparent signal-to-noise (aSNR) and apparent contrast-to-noise ratio (aCNR) (**b**)

No sequence of the bpMRI protocol was rated as non-diagnostic in any category by any rater. All sequences of the bpMRI protocol had a good or excellent overall image quality (averaged median score T2w: 5 [4, 5]; averaged median score b0/800: 4 [4, 5]; averaged median score b1500: 4 [4, 5]), while diagnostic confidence was equally good or excellent (averaged median score T2w: 5 [4, 5]; averaged median score b0/800: 4 [4]; averaged median score b1500: 5 [4, 5]) (Fig. 2a). Separate median scores per rater are shown in Table 3. Interrater agreement between readers was good, with a Cohen’s κ of 0.78 [95% CI: 0.76, 0.81].

**Table 3:** Separate qualitative ratings for both raters

Rater	Category	T2w TSE	DWI b0/800	b1500
1	Artifacts	4 [IQR: 4–4]	4 [IQR: 4–4]	4 [IQR: 4–4]
	Image sharpness	5 [IQR: 4–5]	4 [IQR: 4–5]	3 [IQR: 3–4]
	Lesion conspicuity	5 [IQR: 4–5]	4 [IQR: 3–4]	5 [IQR: 4–5]
	Capsule delineation	4 [IQR: 4–5]	5 [IQR: 4–5]	4 [IQR: 3–4]
	Overall image quality	5 [IQR: 4–5]	4 [IQR: 4–5]	4 [IQR: 4–5]
	Diagnostic confidence	5 [IQR: 4–5]	4 [IQR: 4–4]	5 [IQR: 4–5]
2	Artifacts	4 [IQR: 4–4]	4 [IQR: 4–4]	4 [IQR: 4–4]
	Image sharpness	5 [IQR: 4–5]	4 [IQR: 4–5]	3 [IQR: 3–4]
	Lesion conspicuity	4 [IQR: 4–5]	4 [IQR: 3–4]	5 [IQR: 4–5]
	Capsule delineation	4 [IQR: 4–5]	5 [IQR: 4–5]	4 [IQR: 3–4]
	Overall image quality	5 [IQR: 4–5]	4 [IQR: 4–4]	4 [IQR: 4–4]
	Diagnostic confidence	4 [IQR: 4–5]	4 [IQR: 4–4]	5 [IQR: 4–5]

Variables are shown as median and interquartile range (IQR)

*DWI* diffusion-weighted imaging, *T2w TSE* T2-weighted turbo spin echo

**Fig. 3:**
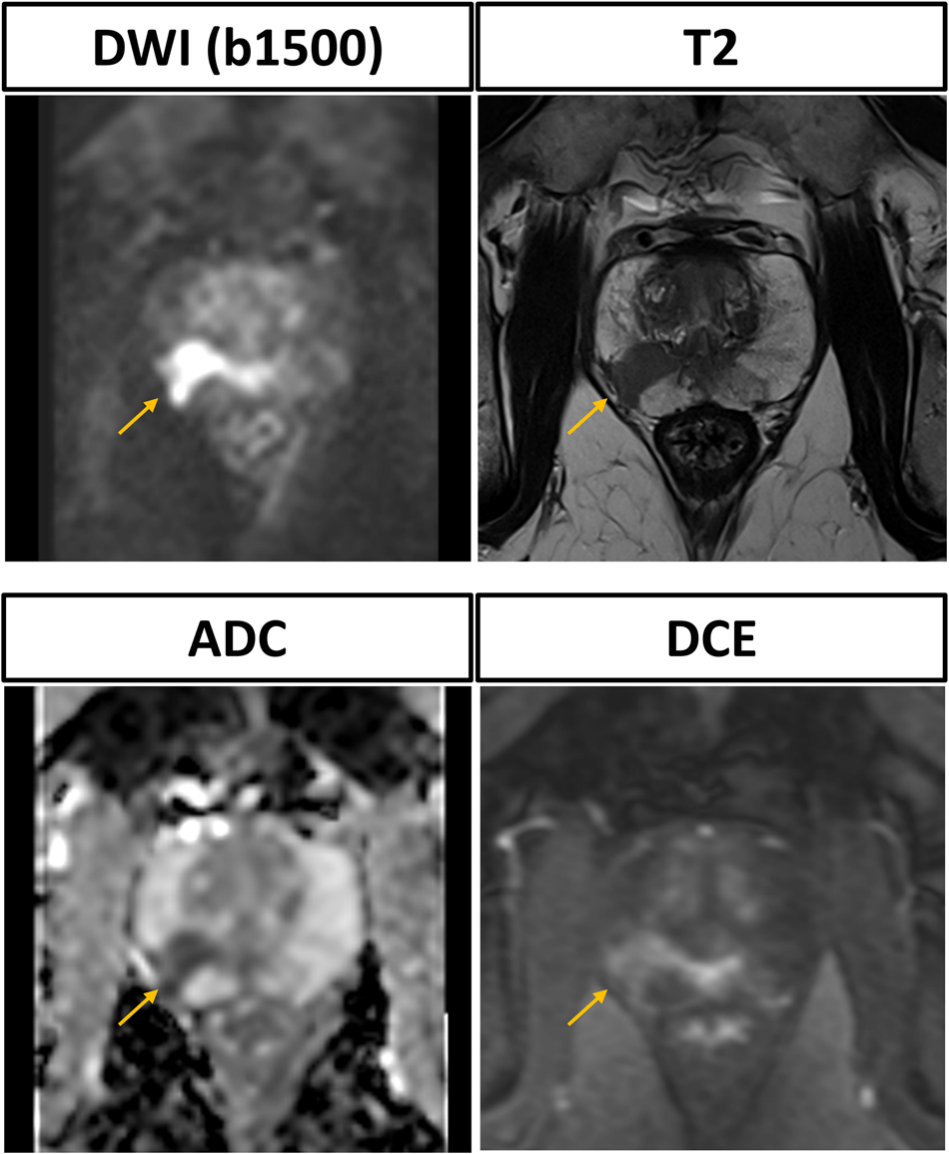
Prostate MRI scan of a 64-year-old male participant with elevated prostate-specific antigen (50 ng/mL), suspicious digital rectal exam and suspicious transrectal ultrasound. The biparametric protocol, consisting of T2-weighted and diffusion-weighted imaging (DWI) with calculation of the apparent diffusion coefficient (ADC) clearly reveals the PI-RADS 5 lesion in the right posterolateral peripheral zone with infiltration of the contralateral posteromedial zone. The dynamic contrast-enhanced sequence shows contemporaneous enhancement of the lesion compared to the normal prostatic tissue. Histopathological analysis after MRI fusion biopsy revealed invasive prostate carcinoma with an International Society of Pathology Grade of 5

**Fig. 4:**
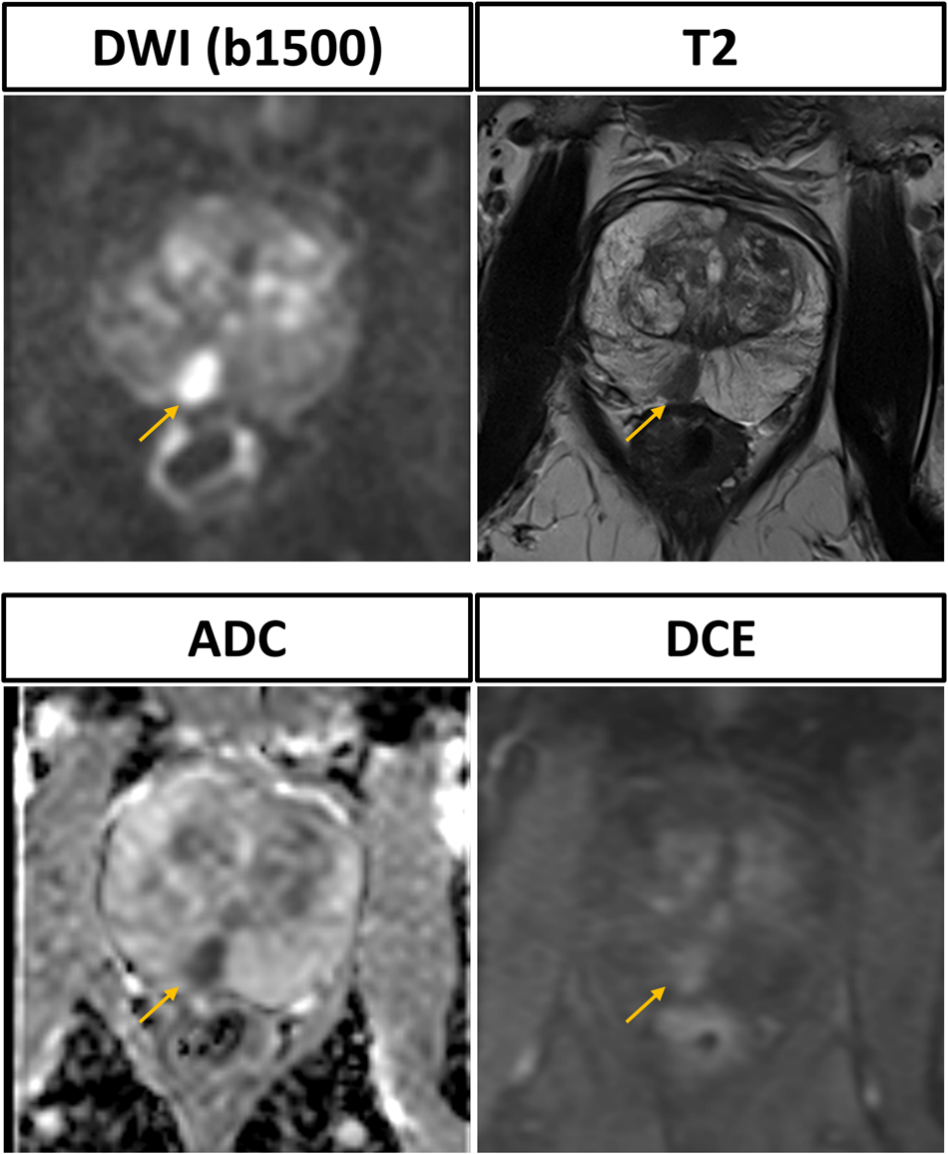
Prostate MRI scan of a 76-year-old male participant with elevated prostate-specific antigen (10 ng/mL) and suspicious transrectal ultrasound. The PI-RADS 4 lesion in the right posteromedial zone is clearly delineated in the T2-weighted (T2w) sequence and shows strong diffusion restriction in diffusion-weighted imaging (DWI) and the corresponding apparent diffusion coefficient (ADC) map. Dynamic contrast enhanced (DCE) shows contemporaneous enhancement. Subsequent MRI fusion biopsy was performed and histopathological analysis confirmed invasive prostate carcinoma with an International Society of Pathology grade of 2

The mean total acquisition time of the bpMRI protocol was reduced to 5 min, 33 ± 21 s compared to the mpMRI protocol with 14 min, 50 ± 42 s. The mean apparent signal-to-noise ratio of the axial T2w sequence was 45.0 ± 7.4, while the mean contrast-to-noise ratio was 40.1 ± 7.1. The mean signal-to-noise and contrast-to-noise ratio of the DWI (b1500) sequence were 9.8 ± 3.0 and 5.3 ± 3.0, respectively (Fig. 2b). Representative images of participants are shown in Figs. 3 and 4.

### Comparison of PI-RADS scores

Agreement of PI-RADS scores for the bpMRI and mpMRI protocols with respect to the whole prostate was excellent for all raters (Cohen’s κ reader 1: 0.95 [95% CI: 0.91, 0.99]; reader 2: 0.95 [95% CI: 0.91, 0.99]; reader 3: 0.94 [95% CI: 0.89, 0.99]). PI-RADS scores for bpMRI and mpMRI protocols were similar and their agreement for all lesions in the peripheral zone and the transition zone was equivalently excellent (Table 4). Overall interrater agreement was good (Fleiss’ κ, 0.86 [95% CI: 0.80, 0.92]). Contingency tables for the correlation of the bpMRI and mpMRI for all three readers are shown in Table 5.

**Table 4:** PI-RADS score agreement between the biparametric and multiparametric MRI protocol

Parameter	Reader 1	Reader 2	Reader 3
Whole prostate			
PI-RADS score bpMRI	2 [IQR: 2–4]	2 [IQR: 2–4]	2 [IQR: 2–4]
PI-RADS score mpMRI	2 [IQR: 2–4]	2 [IQR: 2–4]	2 [IQR: 2–4]
Agreement	0.95 (95% CI: 0.91, 0.99)	0.95 (95% CI: 0.91, 0.99)	0.94 (95% CI: 0.89, 0.99)
Peripheral zone			
PI-RADS score bpMRI	2 [IQR: 2–2]	2 [IQR: 2–2]	2 [IQR: 2–2]
PI-RADS score mpMRI	2 [IQR: 2–2]	2 [IQR: 2–2]	2 [IQR: 2–2]
Agreement	0.96 (95% CI: 0.92, 1.0)	0.96 (95% CI: 0.91, 1.0)	0.94 (95% CI: 0.88, 0.99)
Transition zone			
PI-RADS score bpMRI	3 [IQR: 2–4]	2 [IQR: 2–4]	3 [IQR: 2–4]
PI-RADS score mpMRI	3 [IQR: 2–4]	2 [IQR: 2–4]	3 [IQR: 2–4]
Agreement	0.96 (95% CI: 0.88, 1.0)	0.95 (95% CI: 0.87, 1.0)	1.0 (95% CI: 1.0, 1.0)

PI-RADS scores are reported as median and interquartile range (IQR). Agreement is reported as Cohen’s κ with their respective 95% confidence intervals (CI)

*bpMRI* biparametric MRI, *mpMRI* multiparametric MRI

**Table 5:** Contingency tables of PI-RADS ratings for all readers

Reader 1			bpMRI				
			*n* = 0	*n* = 38	*n* = 8	*n* = 18	*n* = 13
PI-RADS scores (*n* = 77)			1	2	3	4	5
	*n* = 0	1	**0**	0	0	0	0
	*n* = 38	2	0	**38**	0	0	0
mpMRI	*n* = 4	3	0	0	**4**	0	0
	*n* = 22	4	0	0	4	**18**	0
	*n* = 13	5	0	0	0	1	**12**

Reader 2			bpMRI				
			*n* = 0	*n* = 41	*n* = 8	*n* = 15	*n* = 13
PI-RADS scores (*n* = 77)			1	2	3	4	5
	*n* = 0	1	**0**	0	0	0	0
	*n* = 41	2	0	**41**	0	0	0
mpMRI	*n* = 4	3	0	0	**4**	0	0
	*n* = 19	4	0	0	4	**15**	0
	*n* = 13	5	0	0	0	1	**12**

Reader 3			bpMRI				
			*n* = 0	*n* = 38	*n* = 10	*n* = 17	*n* = 12
PI-RADS scores (*n* = 77)			1	2	3	4	5
	*n* = 0	1	**0**	0	0	0	0
	*n* = 38	2	0	**38**	0	0	0
mpMRI	*n* = 4	3	0	0	**4**	0	0
	*n* = 23	4	0	0	6	**17**	0
	*n* = 12	5	0	0	0	0	**12**

If the same PI-RADS score was given for the bpMRI and mpMRI protocol, the corresponding value (number of patients with this PI-RADS score) was written bold.

Data are absolute numbers

*bpMRI* biparametric MRI, *mpMRI* multiparametric MRI, *PI-RADS* Prostate Imaging Reporting and Data System

## Discussion

New technological advancements in ultra-high gradient performance 3.0-T MRI can be used to shorten echo times and therefore accelerate sequence acquisition times (i.e., for diffusion imaging) [16]. Therefore, this study explored the use of a new clinical MRI scanner with a maximum gradient strength of 200 mT/m in combination with commercially available deep learning (DL) reconstruction to investigate the feasibility of a super-fast and high-quality biparametric MRI (bpMRI) of the prostate. This study aimed to compare the assigned Prostate Imaging Reporting and Data System (PI-RADS) scores of this bpMRI protocol with the scores of a standard multiparametric MRI (mpMRI) protocol. A second objective was to assess the image quality of the bpMRI protocol sequences. We found the abbreviated bpMRI protocol to have an excellent PI-RADS agreement with the standard mpMRI protocol, while both T2-weighted (T2w) and diffusion-weighted imaging (DWI) sequences had an excellent overall image quality with a low mean total acquisition time of 5 min and 33 ± 21 s.

In contrast to previous clinical scanners, this MRI system reaches ultra-high gradients of up to 200 mT/m. It should however be noted that these maximum values are not yet fully applicable for full body imaging in clinical routine due to cardiac stimulation limitations, thus the maximum gradient strength in our study was limited to 111 mT/m by the software. However, this gradient strength directly translated to a reduction in echo and repetition times. This becomes particularly clear when comparing the scan parameter adjustments of our sequence made by another 3-Tesla system of the same manufacturer (Siemens MAGNETOM Vida, Siemens Healthineers) after copying the sequence with otherwise identical parameter specifications. For instance, the echo time of the DWI (b1500) sequence could be reduced by 10% to 57 ms and the repetition time by 35% to 3200 ms. Furthermore, in two recent studies [6, 8], in which all qualitative and quantitative parameters were evaluated methodically equivalent by the same raters, the image quality in standard sequences was significantly worse compared to the current study. For instance, in the most recent study [6], the overall image quality of standard non-cartesian T2w sequences was rated as good with a median score of 4 [IQR: 3–4], while the T2w sequence in this study was rated as excellent with a median score of 5 [4, 5]. Equivalently, the aSNR was significantly lower in the standard non-cartesian T2w sequence (25.8 ± 4.6) versus the T2w sequence in the current study (45.0 ± 7.4).

According to the PI-RADS classification [4], a multiparametric MRI of the prostate is mandatory for a thorough evaluation of prostate cancer and consists of T1-weighted, T2-weighted (T2w), DWI and dynamic contrast-enhanced imaging. However, a clear recommendation for the use of primarily biparametric protocols, consisting only of T2w imaging and DWI, is not given by the PI-RADS classification. Several studies already have shown similar accuracy and sensitivity of bpMRI compared to mpMRI [18–21], but the bpMRI protocols investigated in these studies still had long acquisition times of > 10 min due to conventional reconstruction techniques like wavelet transformation [22] or acquisition of several planes for T2w imaging. Furthermore, these studies did not investigate separate b-values in a biparametric approach or achieved both, high image quality and low acquisition times. In contrast to this, we utilized high gradients and slew rates to acquire a fast bpMRI with an axial T2w sequence, a low b-value DWI (b0,800) sequence for computing the apparent diffusion coefficient and a high b-vaIue DWI (b1500) sequence. This resulted in a low total mean acquisition time of 5 min and 33 ± 21 s for the bpMRI versus 14 min, 50 ± 42 s for the mpMRI.

Another advancement and reason for the reduction in acquisition time is the employment of DL denoising and resolution upscaling. While these techniques already have been investigated in T2w imaging of the prostate [6, 23], it has also been shown to be promising for the reconstruction of DWI sequences [24, 25]. Similar to these studies, we found our whole bpMRI protocol to have a mostly good or excellent image quality for both T2w and DWI sequences when reconstructed with this technique, e.g., in image sharpness of the axial T2w sequence or lesion conspicuity of the DWI (b1500) sequence. Quantitative analysis mainly confirmed these findings when compared to literature and showed similar high values for the apparent signal-to-noise ratio and apparent contrast-to-noise ratio in T2w sequences [6], but had slightly lower values for the apparent signal-to-noise ratio and apparent contrast-to-noise ratio in DWI sequences [24], which could be due to small differences in the anatomic region (pathologic versus physiologic) that is ultimately measured.

Finally, we found a high PI-RADS agreement among readers between the bpMRI and mpMRI protocols with changed PI-RADS scores in only 4–6 participants (5–8%). After stratifying assigned PI-RADS scores for the peripheral and transition zones, we found no difference between protocols for the transition zone, while the agreement was slightly lower for the peripheral zone. This is mainly explained by the role of the dynamic contrast-enhanced sequence, as it is only relevant for further evaluation of PI-RADS 3 lesions in the peripheral zone. Additionally, the incidence of PI-RADS 3 lesions in our study was low (bpMRI readings: 8–10 participants [10–13%]), whereas prior bpMRI studies reported higher incidences of > 30% [26, 27]. It remains unclear, if this is coincidently or due to the use of a high gradient MRI scanner with DL reconstruction, as we did not investigate intraindividual comparisons between different scanners.

Our study has some limitations. First, not all participants underwent subsequent biopsy due to refusal of the procedure or low initial PI-RADS scores. Thus, correlation of histopathologic findings with PI-RADS scores and calculation of accuracy of the bpMRI versus the mpMRI was not possible, as it would be strongly biased towards higher PI-RADS scores. Additional studies with biopsies of all participants are needed. Second, the most significant lesions (PI-RADS 3–5) were found in the peripheral zone and were underrepresented in the transition zone. This potentially led to a bias towards the peripheral zone in the evaluation of the bpMRI. However, as DCE is mainly necessary for further assessment of PI-RADS 3 lesions in the peripheral zone, we still had adequate sample sizes that reflect the real world. Third, the study was carried out on a new 3.0-T system with high gradient strength. While this scanner is affordable for large hospitals, smaller hospitals or outpatient centers may have difficulties to buy and establish this scanner, thus it may take a long time for widespread establishment of this technique. Fourth, although MRI acquisition time can be significantly reduced, sufficient time must be allotted for MRI patient preparation. However, productivity could be even more increased by improvements in workflow and modern scheduling software.

In conclusion, we established a high-quality, 5-min biparametric MRI protocol for imaging suspected prostate cancer patients utilizing ultra-high gradients and deep learning reconstructions on a novel MRI scanner. Furthermore, the PI-RADS agreement between the biparametric and the full multiparametric protocol was excellent, thus we firmly believe this technique and this focused examination approach make the detection of prostate cancer more efficient. However, full correlation with histopathological analysis in future studies must be further addressed for valid calculation of diagnostic performance and accuracy.

## Supplementary information

ELECTRONIC SUPPLEMENTARY MATERIAL

## Data Availability

Data reported in this study is available by the corresponding author upon reasonable request.

## Abbreviations

bpMRI: Biparametric magnetic resonance imaging
DWI: Diffusion-weighted imaging
IQR: Interquartile range
mpMRI: Multiparametric magnetic resonance imaging
PI-RADS: Prostate Imaging Reporting and Data System
T2w: T2-weighted
