# Supplementary material for: Ultra-high gradient performance 3-Tesla MRI for super-fast and high-quality prostate imaging: initial experience
Source: Insights Imaging. 2024 Nov 29;15:287. doi: 10.1186/s13244-024-01862-x (PMC11607256; doi:10.1186/s13244-024-01862-x)
Supplement: Supplementary file 1 — ELECTRONIC SUPPLEMENTARY MATERIAL [file 13244_2024_1862_MOESM1_ESM.pdf]

Ultra-high gradient performance 3 Tesla MRI for super-fast and high-quality prostate imaging: Initial experience

ELECTRONIC SUPPLEMENTARY MATERIAL

Table S1: Biopsy results

PI-RADS Score	Performed fusion Biopsy (n=34)	Confirmed malignancy (n=17)	ISUP grade
1 (n=0)	-	-	-
2 (n=36)	5 (14)	2 (40)	Grade 1: 1 (50)<br>Grade 5: 1 (50)
3 (n=10)	4 (40)	1 (25)	Grade 2: 1 (100)
4 (n=19)	16 (84)	10 (63)	Grade 1: 4 (40)<br>Grade 2: 5 (50)<br>Grade 5: 1 (10)
5 (n=12)	9 (75)	8 (89)	Grade 1: 2 (25)<br>Grade 2: 5 (63)<br>Grade 5: 1 (13)

Note.—Dichotomous data is reported as number of participants with percentages in parentheses. ISUP: International Society of Pathology; PI-RADS: Prostate Imaging Reporting and Data System.
